# Tests of Concrete Strength across the Thickness of Industrial Floor Using the Ultrasonic Method with Exponential Spot Heads

**DOI:** 10.3390/ma13092118

**Published:** 2020-05-02

**Authors:** Bohdan Stawiski, Tomasz Kania

**Affiliations:** 1 Faculty of Environmental Engineering and Geodesy, Wrocław University of Environmental and Life Sciences, pl. Grunwaldzki 24, 50-363 Wrocław, Poland; bohdan.stawiski@upwr.edu.pl; 2 Faculty of Civil Engineering, Wrocław University of Science and Technology, Wybrzeże Wyspiańskiego 27, 50-370 Wrocław, Poland

**Keywords:** concrete floors, compressive strength, strength distribution, industrial floors, ultrasound tests

## Abstract

The accepted methods for testing concrete are not favorable for determining its heterogeneity. The interpretation of the compressive strength result as a product of destructive force and cross-section area is burdened with significant understatements. It is assumed erroneously that this is the lowest value of strength at the height of the tested sample. The top layer of concrete floors often crumble, and the strength tested using sclerometric methods does not confirm the concrete class determined using control samples. That is why it is important to test the distribution of compressive strength in a cross-section of concrete industrial floors with special attention to surface top layers. In this study, we present strength tests of borehole material taken from industrial floors using the ultrasonic method with exponential spot heads with a contact surface area of 0.8 mm^2^ and a frequency of 40 kHz. The presented research project anticipated the determination of strength for samples in various cross-sections at the height of elements and destructive strength in the strength testing machine. It was confirmed that for standard and big borehole samples, it is not possible to test the strength of concrete in the top layer of the floor by destructive methods. This can be done using the ultrasonic method. After the analysis, certain types of distributions of strength across concrete floor thickness were chosen from the completed research program. The gradient and anti-gradient of strength were proposed as the new parameters for the evaluation of floor concrete quality.

## 1. Introduction

Composite materials belong to an exceptional group because it is difficult to control grain distribution in the material being formed. The most characteristic example of such a composite is concrete, the most broadly used among them in the construction industry. Tests of this material concern both spatial structure (porosity, tightness, pore, and grain distribution) and its basic parameter, that is, compressive strength. Modifications of this composite aim at the improvement of its homogeneity [1,2,3,4].

**Figure 1:**
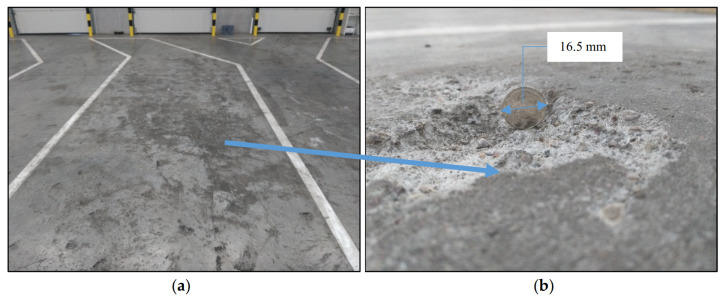
Example of crumbling on the top layer of concrete industrial floor: (**a**) General view; (**b**) close-up of cracked surface.

The industrial floor belongs to those building structures in which concrete should be of the highest quality in the top layer because it is exposed to considerable abrasion, local pressure, impacts, etc. Often, the top layer of the concrete floor quickly crumbles (Figure 1) and strength testing using sclerometric methods does not confirm the concrete class determined on control samples [5].

Strength tests on borehole material of small diameter and height, cut out from various depths, indicate often considerable weakening of concrete in the top layer compared to strength determined on samples cut out from a deeper layer. This problem was noticed in the European standard [6], highlighting that in the top layer, compressive strength can be lower than in the bottom layer by as much as 25%. However, the given value is based on tests of samples from a height not lower than 50 mm. Excessive reduction of samples is not acceptable due to the thickness of the maximum aggregate fraction. Practically, it can be assumed that minimum samples are 50 mm high and before cutting off of the top layer approximately 60 mm high. Compressive strength determined on such samples using the destructive method refers to concrete at a depth of 30 mm from the floor top. Many things indicate that most of the unfavorable phenomena accumulate in this 30 mm small layer. Tests on this type of structure should focus first on this layer. Since in the available scientific literature, there are few test results concerning distributions of compressive strength across the thickness of industrial floors in thin cross-sections taking into account the top layers, the authors of this study undertook this task. Recent articles [7,8,9] shows that acoustic non-destructive methods are suitable for testing concrete strength and heterogeneity. The concrete strength tests presented in this article were performed using the ultrasound method using spot heads. The ultrasonic wave velocities were correlated with the results of destructive tests. The obtained results from tests concerning strength distributions of floors were arranged by types. Additional characteristics of concrete were proposed based on the strength change rate using mathematical bases of quality evaluation.

## 2. Literature Review

In calculation theories, concrete is usually treated as a homogeneous entity, specified often as quasihomogeneous, at least in the macroscopic sense. The question of the actual heterogeneity of concrete remains open for several reasons. The most important reason is that the accepted test methods do not favor recognition of its heterogeneity. According to the binding standard [10], they are conducted on big material samples: cubes with dimensions 150 mm × 150 mm × 150 mm or cylinders with a diameter of 150 and a height of 300 mm, which blur porosity changes at the height of sample elements.

The heterogeneity of strength for elements with a large height dimension, such as pillars and vertical partitions formed in the built-in position, was the subject matter of a publication. Research showed a weakening of concrete in their horizontal cross-sections along with an increase in the measurement height. Tests conducted during the period from 1962 until 1978 in Japan [11] on concrete elements 4 m high formed in the built-in position indicated a 10% to 20% strength increase in the bottom zone and a 10% to 30% strength decrease in the top zone versus middle cross-sections. Giaccio and Alberto Giovambattista [12] showed a 30% reduction of concrete compressive strength in a water dam formed in a vertical position along with an increase in the measurement height. The water draining capacity measured experimentally in concrete used to form these elements was more than 10%. 

Khatib and Mangat showed that the cement pastes formed horizontally can have the pore volume twice as large near the top layer than near the bottom layer. Pores near the top layer are also considerably larger (from 46% to 98% of pores above 0.1 µm) than pores near the bottom layer (from 38% to 80% respectively) [13]. The weakening of concrete structures in top cross-sections along the direction of forming and strengthening in lower cross-sections is effected mainly by: the designed aggregate composition, type and quality of cement, quantity of fly ash, consistency of the concrete mix, use of chemical additions binding water, and the vibration method applied to the formed concrete element. The intensity of water drainage from the concrete mix and the rate of desegregation of its components depends on these factors. This phenomenon is called ‘bleeding’ [14,15,16,17]. It is a spontaneous process that is the result of the difference in densities between the binding agent, aggregate composition, and water. In the technical literature [18,19], depending on the type and method of making the elements, two types of bleeding are distinguished:

- internal, which is characteristic for stocky cross-sections formed in the vertical position,
- superficial, which is observed in the case of laying floors and elements of small thickness in the horizontal position.

Topics regarding strength distribution at the height of stocky cross-sections formed in a vertical position are researched, described, and characterized in the technical and scientific literature. Superficial bleeding, the homogeneity characteristics of near-surface structures, and strength distribution for concrete floors in thin layers are not topics broadly described in the scientific literature. Information about mathematical criteria used to describe strength variability in industrial floors is also missing. Cases of superficial damage to the floors which are the subject of this study are frequent, therefore the authors undertook such research.

## 3. Materials and Test Methods

**Figure 2:**
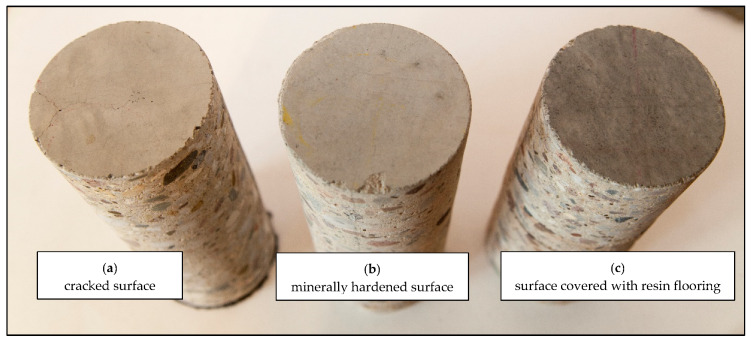
Borehole materials from the tested floors: (**a**) cracked surface; (**b**) hardened surface without damage; (**c**) resin flooring on concrete.

The research project assumed that the analysis would be carried out on samples from borehole materials taken from various regions of the country to avoid any potential material-related errors connected with, for example, the aggregate source. The floors were constructed of concrete class C25/30 and C30/37. Borehole materials with diameters 80 and 100 mm were provided, 6 pieces from each tested floor. They came from floors that were not superficially hardened, from floors with surface hardened using mineral agents and also with resin flooring (Figure 2).

**Figure 3:**
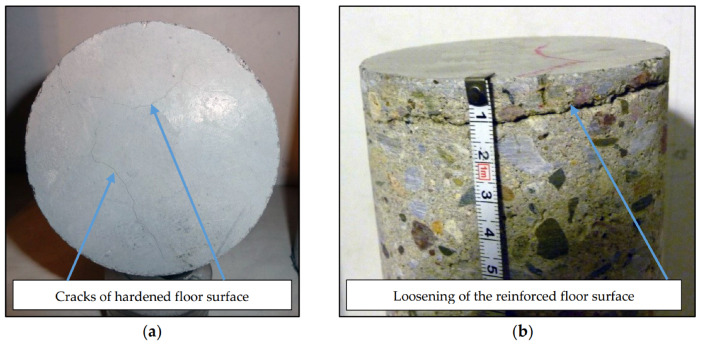
Tested borehole material from floors with a hardened top layer with damage: (**a**) cracked surface; (**b**) loosened surface.

The hardened layer in certain areas of the halls became loosened which is regarded as an emergency condition (Figure 3).

The presented damage indicates substrate hardening of low strength (weak concrete).

The compressive strength of concrete samples in their various cross-sections was determined with the use of the non-destructive method calibrated with the destructive tests of the samples. This type of examination is being widely used for the range of building materials, such as concrete, wood, steel, ceramics, and for the diagnostics of building structures [20,21,22,23,24,25]. Tests were carried out with the use of the ultrasound method based on longitudinal wave velocities [26]. Dependencies between ultrasonic pulse velocity (*UPV*), elastic modulus (*E*), and Poisson’s ratio (*ν*) were researched and described [27,28]. Passing wave velocity (*C_L_*) is proportional to the square root of the dynamic modulus of elasticity (*E_d_*), and inversely proportional to the square root of its density (*ρ*) in Equation (1):

*C_L_* = (*E_d_*/*ρ* ∙ (1 − *ν_d_*)/((1 + *ν_d_*) ∙ (1 + 2*ν_d_*)))^1/2^ (km/s) (1)

**Figure 4:**
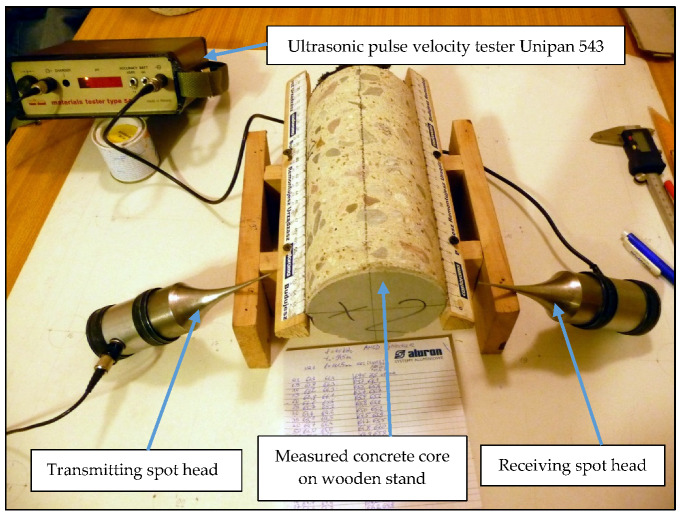
Test stand for measuring ultrasound velocity.

In Equation (1), *ν_d_* is the dynamic Poisson’s ratio. The dependency in Equation (1) applies to homogeneous and isotropic materials. Concrete is a heterogeneous material. High attenuation in concrete limits the *UPV* method to low frequencies (up to 120 kHz). Under this condition, ultrasounds do not interact with most concrete inhomogeneities [29] and it can be regarded as a homogeneous material [30]. Scientific literature shows a strict relationship between concrete compressive strength (*f_c_*) and *UPV* [27,28,30,31,32,33,34,35,36]. This dependency is also described in standards [7,37,38]. Komlos and others [36] stated that UPV non-destructive method of strength testing requires the calibration with the results of destructive tests. Authors research conclusions are the same [36,39]. In the presented research, UPV tests were performed using Unipan 543 tester (company, Warsaw, Poland) and spot heads with frequency 40 kHz. The test stand used to measure ultrasonic wave velocity on the tested concrete borehole material is presented in Figure 4. The results of spot heads testing are presented in the studies [35,39,40].

**Figure 5:**
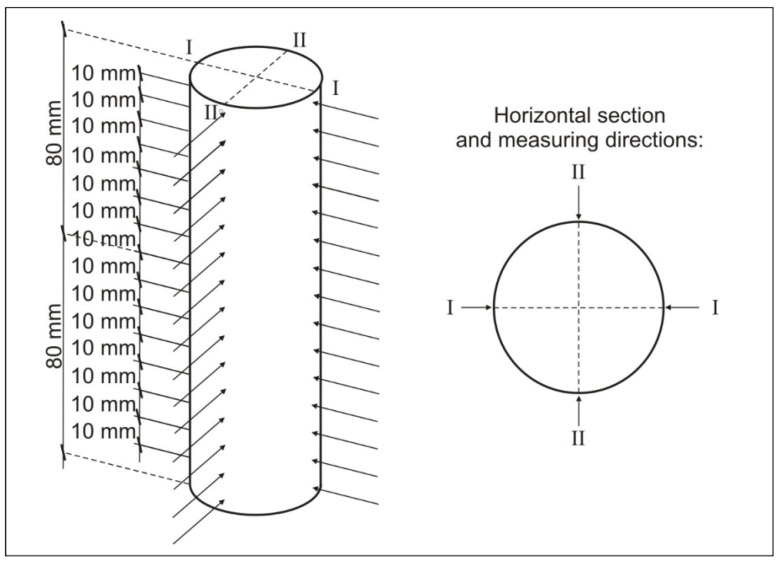
Location of the measurement planes at borehole heights—1st stage of the tests.

At the beginning of the tests, the borehole materials were first cleaned and dried to air-dry moisture according to the standard [6]. Testing cores were stored in laboratory conditions for 14 days before the tests. Then, in the first stage of the tests, ultrasound test planes were marked along the borehole materials (by height) at a distance of 10 mm from each other. In the marked planes, the velocity of ultrasonic wave transmission was tested in two directions, I and II, perpendicular to each other (Figure 5).

**Figure 6:**
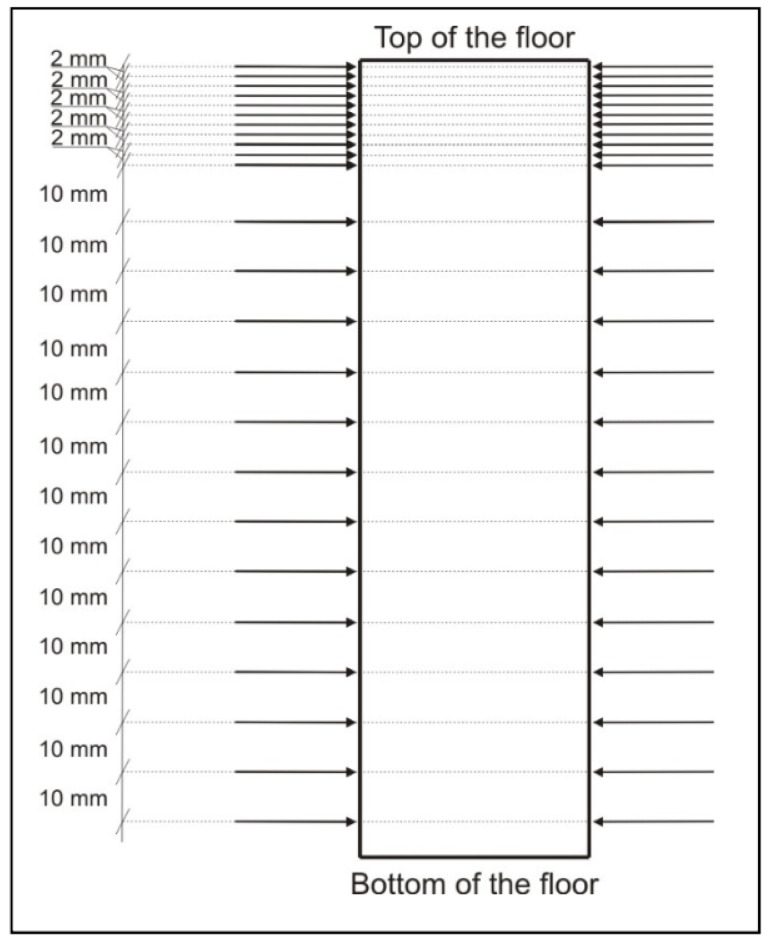
Location of the measurement planes at borehole height—2nd stage of the tests.

In subsequent batches, the top zone of the floor at the thickness of 3–4 cm was examined in more detail. The planes in which measurements were conducted were compacted, at first every 5 mm, and in certain series every 2 mm (Figure 6).

**Figure 7:**
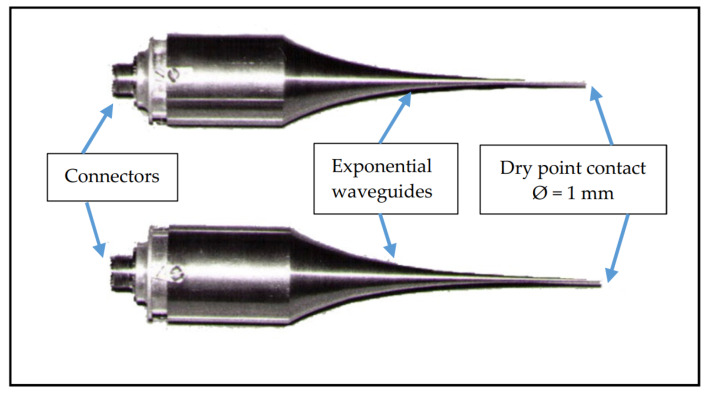
Spot heads with exponential waveguide where the surface area of contact with concrete is 0.8 mm^2^.

Such densely located planes for measurement of the ultrasonic impulse were possible because the diameter of the ends of the spot heads used was only 1 mm (Figure 7).

Boreholes were cut thus obtaining samples with *ϕ = h* (length equal to their diameter). To the pulse velocity determined in the middle of each sample height, compressive strength determined on the strength machine as a relation of destructive force *P* (N) to the surface area of cross-section *A* (mm^2^) of Equation (2) was assigned:

*f_c_* = *P/A* (MPa) (2)

Destructive strength *f_c_* (MPa) and mean ultrasonic wave velocities *C_L_* (km/s) from two testing directions (Figure 5, cross-sections, directions I and II), specified in the middle of the height of the cylindrical sample were the basis for determination of the dependency between ultrasonic pulse velocity and concrete strength for the given series of samples. Scaling curves established hypothetically were used to convert the rate of ultrasound wave in the given cross-section at the borehole height into concrete compression strength in this cross-section. The selection of the dependency *f_c_-C_L_* was made independently for each of the tested concretes taking into account different aggregate, different cement, different additions, and also different conditions of execution not known in detail.

## 4. Results

### 4.1. Calibration of Ultrasound Pulse Velocity-Compression Strength Curves Based on the Strength Machine Tests

After the measurements of ultrasonic pulse velocities of the tested samples, they were cut and tested in uniaxial loading on the strength machine. On that basis, hypothetical scaling curves were chosen.

Scaling Equation (3) was approximated for the measurements of the first concrete type shown in Section 4.2:

*f_c_* = 0.1983*∙C_L_*^4.3081^ (MPa) (3)

The approximation of the scaling curve in Equation (3) was performed based on the results of destructive tests conducted on cylindrical samples with dimensions 10 cm × 10 cm. The results of destructive tests, measured pulse velocities, and strength values calculated using Equation (3) are presented in Table 1.

Scaling curves for the remaining tested boreholes of the floors were approximated in the same way. Scaling curves established hypothetically were used to convert the rate of ultrasound wave in the given cross-section at the borehole height into concrete compression strength in this cross-section.

### 4.2. Tested Types of Strength Distributions in Cross-Section of Concrete Borehole Materials

The tested borehole materials provided an answer to the question of the distribution of concrete strength across the thickness of industrial floors in the tested facilities. Ignoring minor fluctuations of strength at different levels resulting from random positioning of aggregate or small defects in the concrete structure, or errors in measurement of the distance (the head ends got inside surface pores), all results can be grouped according to the summary below.

#### 4.2.1. Concrete “Homogeneous” across Its Thickness with Superficial Weakening

Passing times *t* (µs), wave velocities *C_L_* (km/s), and compressive strengths *f_c_* (MPa) in planes parallel to the surface of the boreholes are presented in Table 2. Calculation of compressive strength from ultrasound wave velocities was done with the use of Equation (3). The borehole materials were taken from an industrial floor 22 cm thick. Results are presented starting with the ordinal number 1 (5 mm from the top of the floor) in the direction of the bottom of the floor.

**Figure 8:**
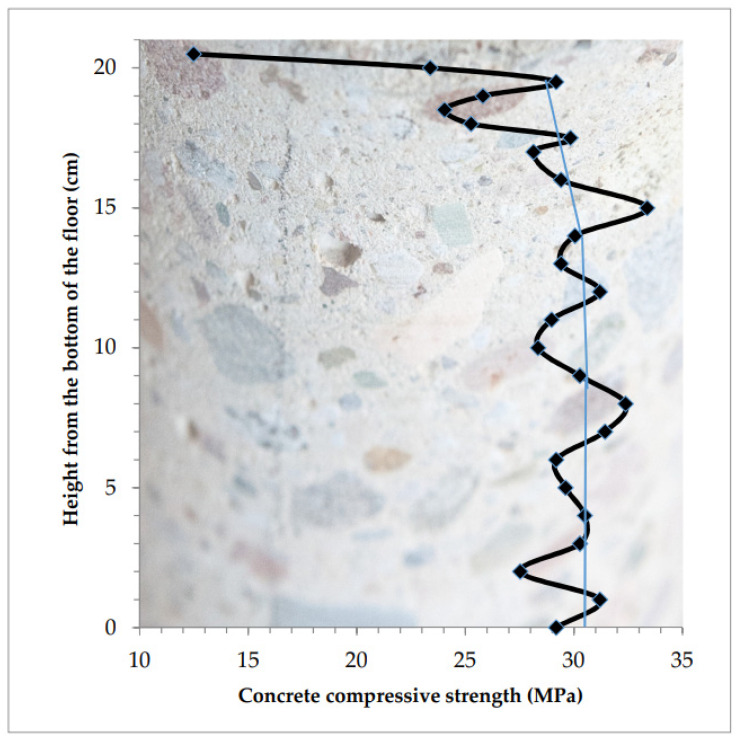
Concrete “homogeneous” across the floor thickness, only the thin top layer is considerably weakened.

Distribution of compressive strength in the tested concrete at the sample height is presented in Figure 8.

Fluctuations across the average strength values (marked with the thin blue line in Figure 8) were relatively small. Its strong decrease begins at 19 cm from the bottom. Concrete is homogeneous across the floor thickness, only the top layer, approximately 10–20 mm, was weakened. The compressive strength of concrete in the top layer of the floor drops from the value of 25–30 MPa down to the value of 12 MPa at the measurement height of 10 mm. 

#### 4.2.2. “Homogeneous” Concrete with Weakening across the Top Layer 30–50 mm Thick

Calculation of compressive strength from ultrasound wave velocities was done with the use of Equation (3). The borehole materials were taken from an industrial floor 21 cm thick. Results are presented in Table 3 starting with the ordinal number 1 (5 mm from the top of the floor) in the direction of the bottom of the floor.

**Figure 9:**
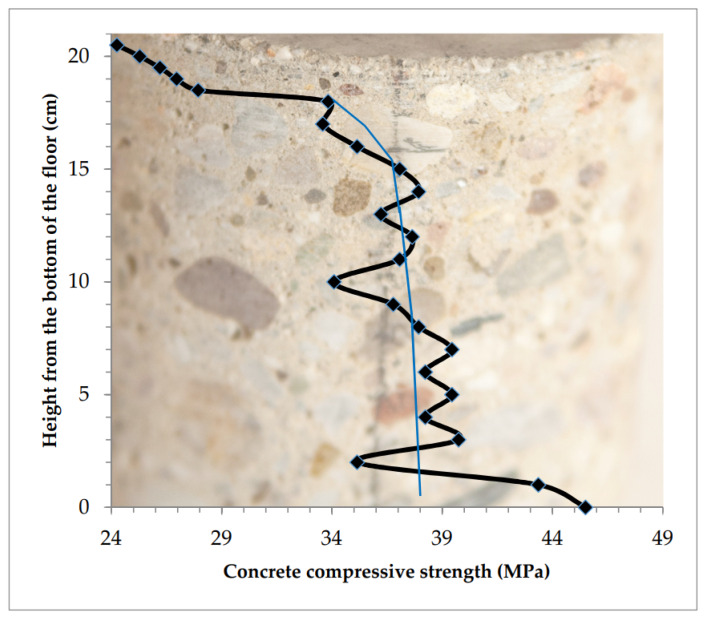
Concrete across the thickness of the floor relatively homogeneous, weakened thick top layer.

The distribution of compressive strength for the tested concrete at the height of the sample is presented in Figure 9.

The concrete was relatively homogeneous across the floor thickness. The weakened top layer was 30–50 mm thick. Compressive strength in this layer of concrete drops from the value of 34–40 MPa down to the value of 24 MPa in the near-surface cross-section.

The bottom layer of the sample was significantly strengthened. Measurements made at a height of 2 mm from the bottom of the sample showed an increase in strength to 46 MPa from 35–40 MPa in close layers above.

#### 4.2.3. Concrete Strength Is Changing across Entire Section with a Quick Weakening of the Top Layer

The borehole materials were taken from an industrial floor 15 cm thick. Calculation of compressive strength from ultrasound wave velocities was done with the use of Equation (4).

*f_c_* = 0.123*∙C_L_*^4.3081^ (MPa) (4)

Results are presented in Table 4, starting with the ordinal number 1 (5 mm from the top of the floor) in the direction of the bottom of the floor.

**Figure 10:**
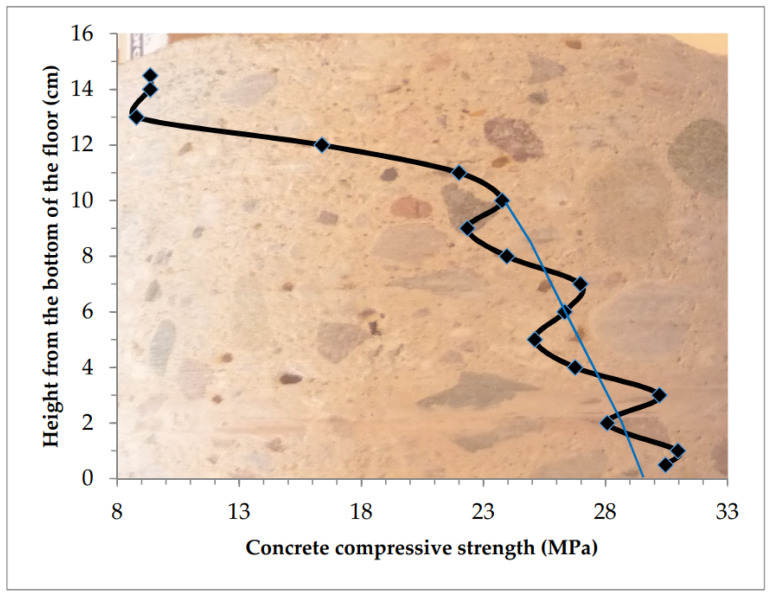
The change of concrete strength across the floor thickness was continuous. The weakened top layer was very thick.

The concrete across the floor thickness changed its strength along with the change in the measurement plane. The top layer with a high thickness (up to 50 mm) became weakened very quickly in the direction of the floor top. The distribution of compressive strength for the tested concrete at the height of the sample is presented in Figure 10.

#### 4.2.4. Concrete Reinforced Superficially to the Expected Value Using the Mineral Powder

To improve the hardness of the top layer of the floor, powders made of hardening materials based on a cement binding agent and strong filler were used. If the strength of the hardened concrete had characteristics in the vertical cross-section similar to the one described in Section 4.2.1 (only a thin 10–15 mm top layer of the floor was weakened), a correctly made hardening layer may balance the shortage of strength. The floor will achieve the designed quality on its top layer. In the presented example top layer of concrete floor has been reinforced using mineral powder to the average level in the vertical cross-section, in an expected way. After tests of the borehole samples of the examined floor on the strength testing machine, the scaling curve described using Equation (3) was adjusted. 

The result of measurements regarding ultrasonic wave velocity and compressive strength in the sample’s cross-section is presented in Table 5.

**Figure 11:**
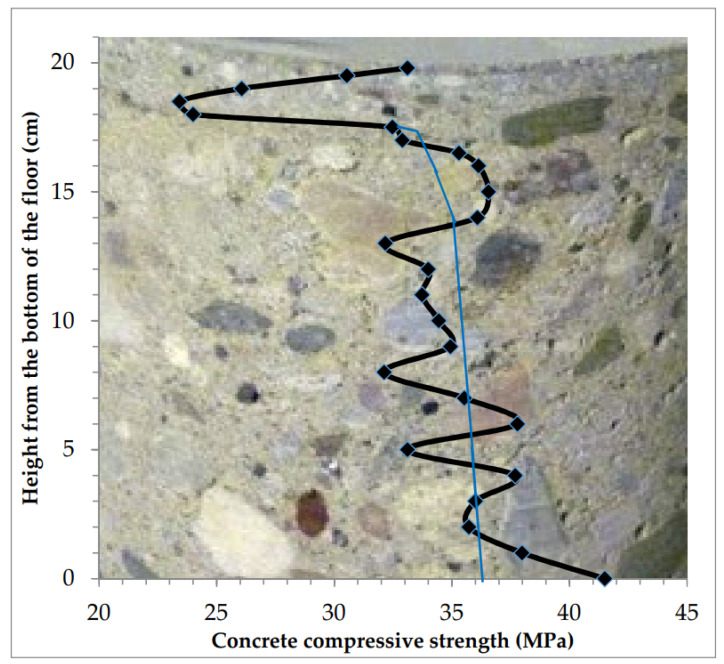
The strength of the floor reinforced superficially to the expected value using the mineral powder.

Distribution of strength in cross-section of the sample taken from the floor with the hardening layer made as expected (Table 5) is presented in Figure 11.

The superficially weakened floor, with the compressive strength of 25–30 MPa, strengthened in the near-surface zone up to the designed value of 30 MPa. Repair efficiency depends on the weakest cross-section under the strengthened layer and its ability to transfer shear stress arising because of the shrinkage of the strengthened layer. In the lower zone of the sample (2 mm from the bottom of the floor) the concrete was strengthened to a value of over 40 MPa. The concrete strengthening zone was about 20 mm thick.

#### 4.2.5. Concrete Floors under Emergency Conditions with Top Layer Hardened using Mineral Powder

As far as concrete floors strengthened (and hardened) using the mineral powder under emergency conditions are concerned, three types of strength distribution were analyzed:

- the case of strengthening the top layer with too small a quantity of mineral powder,
- the case of strengthening the floor with too weak a top layer of concrete,
- the case of excessive strengthening of the top layer of the floor.

Table 6 presents the results of measurements concerning ultrasonic wave velocity in cross-section of the floor strengthened with too small a quantity of mineral powder. The borehole materials were taken from a floor 16.5 cm thick. Calculation of compressive strength from ultrasound wave velocities was done with the use of Equation (5):

*f_c_* = 6.147*∙C_L_*^2^ − 18.172*∙C_L_* + 10.786 (MPa) (5)

**Figure 12:**
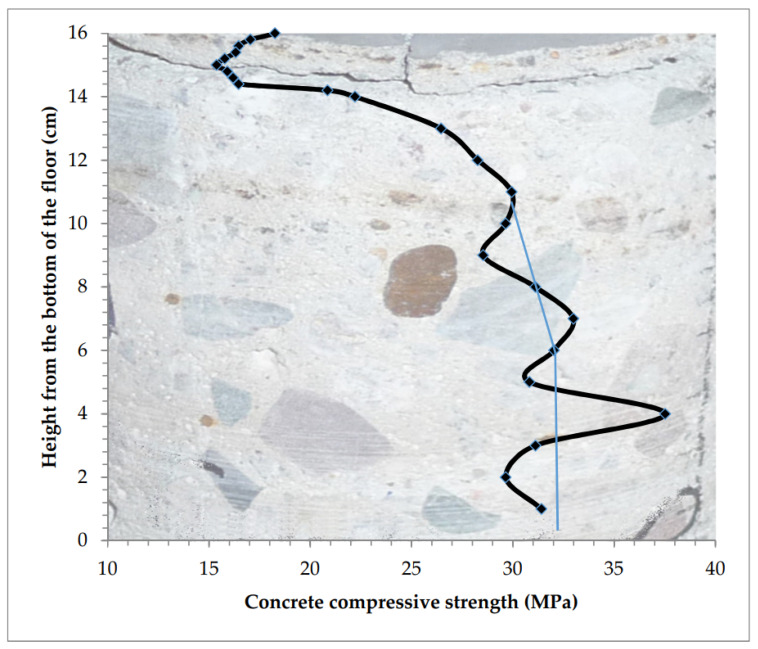
Too weak ineffective strengthening of the industrial floor.

Distribution of strength in cross-section of a sample taken from the floor in which too small a quantity of hardening powder was used is presented in Figure 12.

In this case, the compressive strength drops from a value close to 30 MPa at a depth of 5 cm from the top of the floor to 16 MPa in the zone close to the surface of destruction. The destruction occurred in the weakest layer of concrete, where strength reaches a value of not more than 16.3 MPa. In the reinforced surface plane, the strength increases to 19 MPa at a depth of 3 mm from the top of the floor. The mineral strengthening of the floor, in this case, should reach a depth of 25–30 mm, at which the concrete strength was at a minimum level of 20–25 N/mm^2^.

The strengthening will be ineffective also in the case in which concrete below the zone affected by the mineral powder is too weak and the entire strengthened layer is loosened. Measurements of ultrasonic wave velocity in cross-section of a sample taken from such a floor are presented in Table 7. The dependency between wave velocity *C_L_* and strength *f_c_* described using Equation (6) was used for calculations of the distribution of strength in the sample’s cross-section.

*f_c_* = 112.880*∙C_L_*^2^ − 379.850*∙C_L_* + 324.7 (MPa) (6)

**Figure 13:**
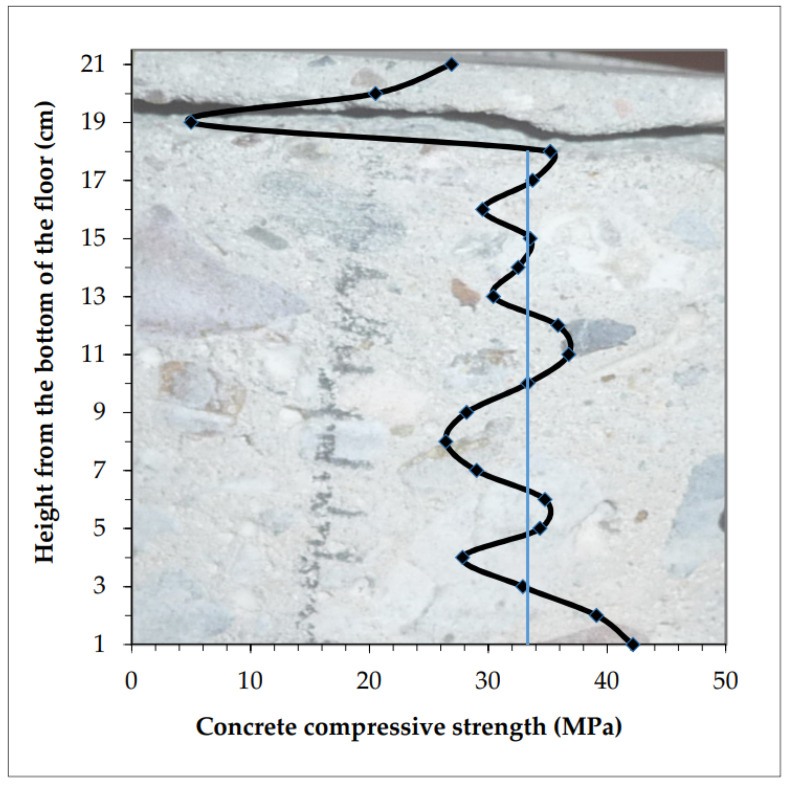
Strengthening that was ineffective because the concrete under the hardening layer had a very low strength of approximately 5 MPa.

The distribution of strength in cross-section of the tested sample is presented in Figure 13.

In this situation, the strengthening was ineffective because directly under the mineralized layer, with a strength of 20 MPa, the concrete was very weak, its strength decreases down to the value of approximately 5 MPa near the surface of destruction, and then increases up to the designed value of 30 MPa.

The floor reaches the highest strength value at the last measuring point, 1 cm deep from the bottom edge. The reinforcement was even at a thickness of 4 cm from the bottom, where the measured strength was 28 MPa and reaches 40 MPa in the plane at the bottom of the sample. The effect of concrete strengthening in the lower planes of industrial floors (Figure 9, Figure 11 and Figure 13) usually results from aggregate segregation, which occurs in the process of vibrating the concrete mix in gravitational field forces.

A similar effect of damage to the hardened floor occurs if excessive powder is used. Measurements regarding ultrasonic wave velocity for a borehole material from such a floor are presented in Table 8. The calibrated dependency of the approximation described using Equation (7) was used for calculation of compressive strength in the cross-section of the sample.

*f_c_* = 24.780*∙C_L_* − 33.600 (MPa) (7)

**Figure 14:**
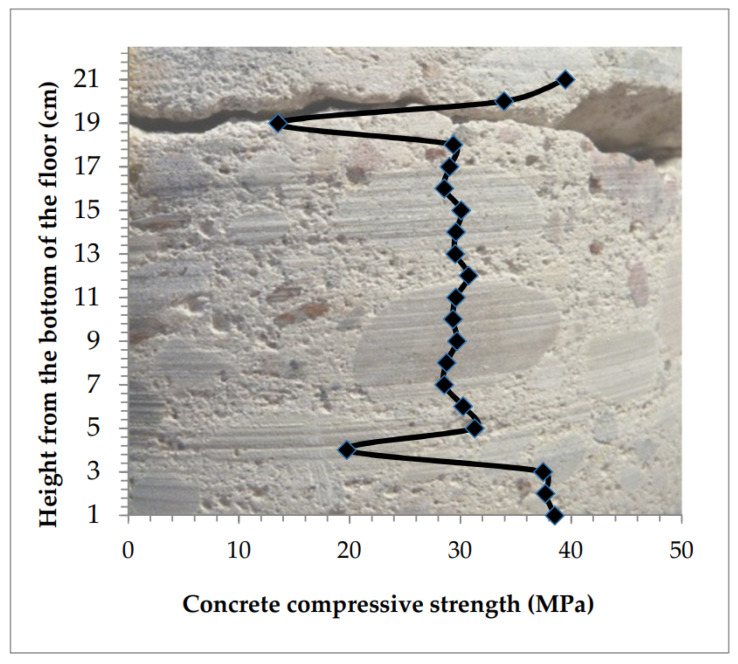
Homogeneous concrete across the thickness of the floor was weakened in the top zone and was strengthened excessively (strength growth from 13 to 40 MPa).

The distribution of strength in the cross-section of the tested sample is presented in Figure 14.

**Figure 15:**
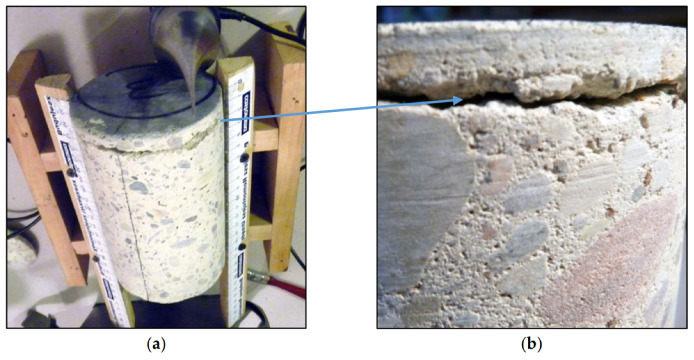
Loosening of the floor from the weak concrete; (**a**) view of the entire sample; (**b**) close-up to the loosened zone.

In this case, the strength of the top layer exceeded the designed value. The measured strength of the borehole material in the middle of the sample’s height has a value of approximately 30 MPa. Considering the possible weakening in the upper zone, the concrete was excessively strengthened with mineral powder. After strengthening, in the near-surface zone, the strength value increased up to 40 MPa, and below the strengthened zone it drops down to the value of 13.5 MPa. In the destruction zone, in which it was impossible to make a measurement, the value of strength was lower than 13.5 MPa—a value measured from its nearest surface (2 cm from the top of the floor). Considerable shrinkage in the place of contact between the layer with increased strength and the weakened layer lead to loosening (Figure 14 and Figure 15).

The various situations involving floor concrete weakening in the top layer shown above, as well as successful and unsuccessful strengthening using hardening powder, explain the causes of the defects and the effects of the ‘repairs’. The examination of concrete strength distribution across the thickness of the floor was possible thanks to the applied ultrasound method with spot heads.

The interpretation of the results obtained from tests of concrete strength distributions across the thickness of the floor presented above is satisfactory, however, no reference documents or admissible values regarding strength variability in a cross-section of concrete slab elements are available concerning this. The subsequent section presents a proposal of additional criteria for assessing concrete in floors and an attempt to indicate mathematical criteria ensuring the basis for evaluation of strength changes across the thickness of the tested concrete layer based on the strength values and the rate of change of strength values obtained from measurements of ultrasonic wave velocities.

### 4.3. Proposal for Evaluating Concrete Quality in the Floor Based on Its Strength Gradient and Anti-Gradient

The gradient of the scalar strength field indicates the direction of the quickest growth of strengths in individual points. The modulus (that is ‘length’) of each vector in such a field is equal to the rate of strength field growth in the given direction. The vector opposite to the gradient is sometimes called an anti-gradient. The strength gradient is a vector value and its unit in the SI system is pascal per meter (Pa/m). Due to the range of the measured strength gradient values for industrial floors, the unit MPa/cm is used for the description of them.

In the considered case of the strength field test, you can refer to a vertical strength gradient that corresponds to a strength change in line with the distance from the bottom of the slab (with the height of the measurement plane). The strength gradient between two measurement planes 1 and 2 can be expressed using Equation (8):

∇*f_c_* = (*f_c,2_* − *f_c,1_*)/(*Z*_2_ − *Z*_1_) (MPa/cm) (8)

where *f_c,2_* is concrete strength in plane 2 (closer to the top surface of the slab); *f_c,1_* is concrete strength in plane 1 (closer to the bottom surface of the slab); *Z*_2_—distance of the second measurement plane from the bottom of the slab; *Z*_1_—distance of the first measurement plane from the bottom of the slab.

**Figure 16:**
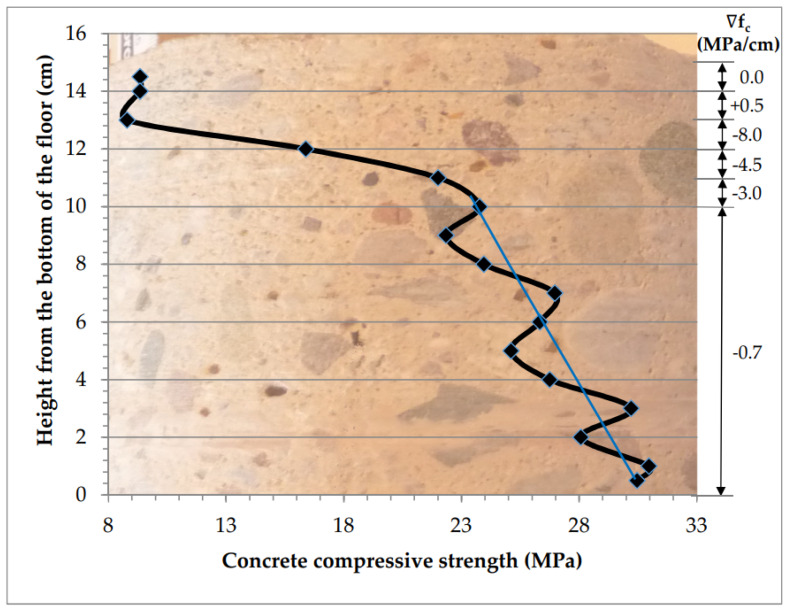
Concrete strength gradients at various floor depths.

Figure 16 shows the calculated gradients between various measurement planes for the borehole material for which measurement results are presented in Table 4.

At a depth of 0 to 10 cm counting from the bottom of the slab, ∇*f_c_* is 0.7 MPa/cm apart from local fluctuations, and in layers located closer to the top surface it changes quickly (−3.0; −4.5; −8.0 MPa/cm). In the zone of powder-based hardening, the strength begins to increase (∇*f_c_* = +0.5 MPa/cm). Changes in the strength of concrete in the areas of fluctuation around the average value (marked with a blue line in Figure 16) are related to the arrangement of grains on the path of ultrasound waves in concrete and are not the subject of consideration. A zone in which there is a rapid decrease or increase in strength is analyzed, e.g., after strengthening and applied to a thin top layer, 20 to 50 mm, rarely thicker. When the anti-gradient is large, the decrease in concrete strength in the surface layer is very large and the floor will need repair. It is proposed to test the gradient in the following variants:

- ∇*f_c,min_*—from the level of the beginning of a rapid decrease in strength (as above) upwards, to the zone of strengthening (increase in strength value) in the case of hardened floors or to the floor surface when there is no strengthening zone;
- ∇*f_c min,10 mm_*—on a thickness of 10 mm up from the place of onset of a rapid decrease in strength in the near-surface area (20–50 mm from the top of the floor);
- ∇*f_c,max_*—gradient of strength increase in the hardening zone, relevant to the floors treated with a surface hardening agent.

A summary showing described strength gradient parameters together with measured extreme and mean values of compressive strength of tested samples is presented in Table 9.

Apart from the minimum, mean, and maximum values of compressive strength in cross-section of industrial floors, the strength variability dynamics can be, in this case, described using the value of strength gradient and anti-gradient, which is especially important for the distribution of strength for near-surface concrete layers. The floor should be made using concrete of a specified class and with strength variability gradient at as low a level as possible, guaranteeing that its performance features are maintained during use. The quality of concrete in an industrial floor can be in this case specified using not one but two parameters. The presented examples of strength distribution in the floors indicate that crumpling and cracking in industrial floors used in a standard way usually occur in the following cases:

- if their minimum compressive strength measured in cross-section is considerably lower than the designed value;
- if their compressive strength in the surface zone is lower than its service load;
- if the strength gradient for the top, hardened layer exceeds the value of shear strength of concrete. Shrinkage in the place of contact between the level of concrete with increased and weakened strength leads to loosening.

Presented measurements of concrete floors require further research, also with the use of different measurement tools. A discussion of a wide pool of strength distribution results is needed, based on which it may be possible to determine the limit values of the measured concrete strength gradients that can be used to standardize the quality assessment of industrial concrete floors. In the future, it is also planned to carry out measurements of ultrasonic wave velocity and strength distribution in the different concrete constructions formed horizontally to compare them with the results presented in this article. The obtained values of reduced compressive strength in the upper zone of concrete elements are also important in the case of bent reinforced concrete slabs, in which it is advisable that the top zone is not weakened.

## 5. Conclusions

Despite meeting the requirements for industrial floors tested by the standard method, local fluctuations in compressive strength values in surface cross-sections are a frequent reason for their failure conditions.

In industrial floors made of concrete without surface hardening, the weakest layer is the top layer 10–50 mm thick. The strength of such thin layers can be tested using the ultrasound method with spot head on borehole materials taken from the structure.

Determination of the distribution and dynamics of concrete compressive strength changes throughout the entire cross-section allows, for example, for the establishing of the thickness of the layer which must be removed during repair of the floor. The reconstructed layer must be laid on concrete having the necessary strength (typically f_c_ ≥ 20 MPa).

Concrete floors analyzed in the article can be divided into three types based on the curve showing the distribution of strength across their thickness. In this case, the one-parameter evaluation of concrete in the floor, based on the standard destructive compressive strength tests, is not sufficient. The addition of a parameter, which is a strength gradient, was proposed. Floor concretes can be controlled based on the tests of concrete compressive strength class and its gradients across the floor thickness.

## Figures and Tables

**Table 1:** Results of uniaxial destructive tests, measured pulse velocities, and strength values calculated with the use of the chosen hypothetical scaling curve.

Ordinal Number	Ultrasound Longitudinal Wave Velocity *C_L_* (km/s)	Compression Strength (MPa)		Difference *f_c_* from Equation (3)—*f_c, Ø10/10_* (MPa)
		Destructive Test	*f_c_* from Equation (3) (0.1983∙*C_L_*^4.3081^)	
		*f_c, Ø10/10_*		
1	3.460	40.24	41.66	1.42
2	3.459	43.22	41.61	−1.61
3	3.404	39.60	38.83	−0.77
4	3.372	38.11	37.28	−0.83
5	3.362	37.55	36.81	−0.74
6	3.288	32.88	33.44	0.56
7	3.282	34.02	33.18	−0.84
8	3.191	30.99	29.40	−1.59
9	3.190	31.77	29.36	−2.41
10	3.172	28.17	28.65	0.48
11	3.168	28.42	28.49	0.07
12	3.167	28.64	28.46	−0.18
13	3.108	27.12	26.24	−0.88
14	3.092	25.16	25.66	0.50
15	3.078	25.21	25.17	−0.04
16	3.07	25.22	24.89	−0.33
17	3.02	23.38	23.19	−0.19
18	2.838	17.22	17.74	0.52
19	2.742	14.62	15.30	0.68
Mean value	3.182	30.081	29.755	−0.33

**Table 2:** Results of concrete ultrasound velocity test in borehole No. 1.

Ordinal Number	Height from the Bottom of the Floor (cm)	Ultrasound Netto Passing Time in Direction I-I *t_n I-I_* (μs)	Ultrasound Netto Passing Time in Direction II-II *t_n II-II_* (μs)	Mean Ultrasound Netto Passing Time *t_n_* (μs)	Ultrasound Wave Velocity *C_L_* (km/s)	Concrete Compression Strength *f_c_* (MPa)
1	21.5	35.3	36.2	35.75	2.615	12.47
2	21.0	29.8	32.0	30.90	3.026	23.39
3	20.5	29.4	29.3	29.35	3.186	29.20
4	20.0	30.2	30.2	30.20	3.096	25.81
5	19.5	30.5	30.9	30.70	3.046	24.06
6	19.0	30.1	30.6	30.35	3.081	25.27
7	18.5	28.6	29.8	29.20	3.202	29.84
8	18.0	29.5	29.7	29.60	3.159	28.15
9	17.0	29.4	29.2	29.30	3.191	29.40
10	16.0	28.5	28.4	28.45	3.286	33.36
11	15.0	28.4	29.9	29.15	3.208	30.08
12	14.0	28.8	29.8	29.30	3.191	29.40
13	13.0	29.1	28.7	28.90	3.235	31.18
14	12.0	29.4	29.4	29.40	3.180	28.96
15	11.0	29.2	29.9	29.55	3.164	28.34
16	10.0	28.5	29.7	29.10	3.213	30.28
17	9.0	28.5	28.8	28.65	3.264	32.40
18	8.0	28.3	29.4	28.85	3.241	31.43
19	7.0	29.2	29.5	29.35	3.186	29.20
20	6.0	28.3	30.2	29.25	3.197	29.63
21	5.0	28.8	29.3	29.05	3.219	30.52
22	4.0	29.0	29.2	29.10	3.213	30.28
23	3.0	29.7	29.8	29.75	3.143	27.54
24	2.0	29.2	28.6	28.90	3.235	31.18
25	1.0	29.5	29.2	29.35	3.186	29.20

**Table 3:** Results of concrete ultrasound velocity test in borehole No. 2.

Ordinal Number	Height from the Bottom of the Floor (cm)	Ultrasound Netto Passing Time in Direction I-I *t_n I-I_*(μs)	Ultrasound Netto Passing Time in Direction II-II *t_n II-II_* (μs)	Mean Ultrasound Netto Passing Time *t_n_* (μs)	Ultrasound Wave Velocity *C_L_* (km/s)	Concrete Compression Strength *f_c_* (MPa)
1	20.5	31.3	30.5	30.90	3.040	24.24
2	20.0	31.1	30.1	30.60	3.070	25.28
3	19.5	30.4	30.3	30.35	3.096	26.19
4	19.0	29.6	30.7	30.15	3.116	26.95
5	18.5	29.7	30.1	29.90	3.142	27.94
6	18.0	28.4	28.8	28.60	3.285	33.83
7	17.0	28.4	28.9	28.65	3.279	33.58
8	16.0	28.4	28.3	28.35	3.314	35.14
9	15.0	27.6	28.4	28.00	3.355	37.07
10	14.0	27.6	28.1	27.85	3.373	37.94
11	13.0	28.0	28.3	28.15	3.337	36.22
12	12.0	27.8	28.0	27.90	3.367	37.64
13	11.0	28.0	28.0	28.00	3.355	37.07
14	10.0	28.6	28.5	28.55	3.291	34.09
15	9.0	27.6	28.5	28.05	3.349	36.78
16	8.0	27.4	28.3	27.85	3.373	37.94
17	7.0	27.6	27.6	27.60	3.404	39.44
18	6.0	27.7	27.9	27.80	3.379	38.23
19	5.0	27.4	27.8	27.60	3.404	39.44
20	4.0	27.7	27.9	27.80	3.379	38.23
21	3.0	27.6	27.5	27.55	3.410	39.75
22	2.0	28.1	28.6	28.35	3.314	35.14
23	1.0	27.4	26.6	27.00	3.480	43.35
24	0.2	27.4	26.0	26.70	3.519	45.49

**Table 4:** Results of concrete ultrasound velocity test in borehole No. 3.

Ordinal Number	Height from the Bottom of the Floor (cm)	Ultrasound Netto Passing Time in Direction I-I *t_n I-I_* (µs)	Ultrasound Netto Passing Time in Direction II-II *t_n II-II_* (µs)	Mean Ultrasound Netto Passing Time *t_n_* (µs)	Ultrasound Wave Velocity *C_L_* (km/s)	Concrete Compression Strength *f_c_* (MPa)
1	14.5	34.2	34.6	34.40	2.733	9.35
2	14.0	34.4	34.4	34.40	2.733	9.35
3	13.0	34.0	35.8	34.90	2.693	8.78
4	12.0	29.7	30.7	30.20	3.113	16.38
5	11.0	28.2	28.2	28.20	3.333	22.00
6	10.0	26.9	28.5	27.70	3.394	23.77
7	9.0	29.2	27.0	28.10	3.345	22.34
8	8.0	28.6	26.7	27.65	3.400	23.95
9	7.0	27.2	26.6	26.90	3.494	26.97
10	6.0	26.9	27.2	27.05	3.475	26.33
11	5.0	27.5	27.2	27.35	3.437	25.11
12	4.0	27.8	26.1	26.95	3.488	26.75
13	3.0	25.9	26.5	26.20	3.588	30.21
14	2.0	26.4	26.9	26.65	3.527	28.07
15	1.0	25.8	26.3	26.05	3.608	30.97
16	0.5	25.7	26.6	26.15	3.595	30.46

**Table 5:** Results of concrete ultrasound velocity test in borehole No. 4.

Ordinal Number	Height from the Bottom of the Floor (cm)	Ultrasound Netto Passing Time in Direction I-I *t_n I-I_* (µs)	Ultrasound Netto Passing Time in Direction II-II *t_n II-II_* (µs)	Mean Ultrasound Netto Passing Time *t_n_* (µs)	Ultrasound Wave Velocity *C_L_* (km/s)	Concrete Compression Strength *f_c_* (MPa)
1	19.8	28.5	28.8	28.65	3.274	32.83
2	19.5	28.7	29.7	29.20	3.213	30.28
3	19.0	29.6	31.0	30.30	3.097	25.84
4	18.5	29.8	32.4	31.10	3.021	23.22
5	18.0	29.4	32.5	30.95	3.038	23.79
6	17.5	28.0	29.6	28.80	3.259	32.19
7	17.0	28.2	29.2	28.70	3.269	32.62
8	16.5	27.5	29.0	28.25	3.323	35.00
9	16.0	27.3	28.9	28.10	3.341	35.83
10	15.0	27.8	28.2	28.00	3.350	36.25
11	14.0	27.4	28.8	28.10	3.340	35.78
12	13.0	28.5	29.2	28.85	3.252	31.89
13	12.0	27.7	29.3	28.50	3.294	33.71
14	11.0	27.7	29.4	28.55	3.288	33.44
15	10.0	27.9	28.9	28.40	3.304	34.15
16	9.0	28.0	28.6	28.30	3.315	34.64
17	8.0	28.9	28.8	28.85	3.251	31.85
18	7.0	28.8	27.6	28.20	3.328	35.23
19	6.0	28.4	27.2	27.80	3.376	37.47
20	5.0	29.0	28.3	28.65	3.274	32.83
21	4.0	27.8	27.8	27.80	3.374	37.38
22	3.0	28.0	28.2	28.10	3.338	35.69
23	2.0	28.0	28.3	28.15	3.332	35.42
24	1.0	27.7	27.8	27.75	3.380	37.67
25	0.2	26.6	27.8	27.20	3.450	41.14

**Table 6:** Results of concrete ultrasound velocity test in borehole No. 5.

Ordinal Number	Height from the Bottom of the Floor (cm)	Ultrasound Netto Passing Time in Direction I-I *t_n I-I_* (µs)	Ultrasound Netto Passing Time in Direction II-II *t_n II-II_* (µs)	Mean Ultrasound Netto Passing Time *t_n_* (µs)	Ultrasound Wave Velocity *C_L_* (km/s)	Concrete Compression Strength *f_c_* (MPa)
1	16.2	30.3	28.3	29.30	3.3560	19.03
2	16.0	30.0	28.8	29.40	3.3450	18.78
3	15.8	30.4	29.2	29.80	3.3000	17.76
4	15.6	31.0	29.0	30.00	3.2780	17.27
5	15.4	30.9	29.2	30.05	3.2730	17.16
6	15.2	30.9	29.6	30.25	3.2510	16.68
7	15.0	31.1	29.7	30.40	3.2350	16.33
8	14.8	30.6	29.8	30.20	3.2560	16.79
9	14.6	30.8	29.4	30.10	3.2670	17.03
10	14.4	30.2	29.8	30.00	3.2780	17.27
11	14.2	29.0	28.3	28.65	3.4320	20.82
12	14.0	28.6	28.0	28.30	3.4750	21.87
13	13.0	27.2	27.5	27.35	3.5960	24.93
14	12.0	27.0	27.0	27.00	3.6420	26.14
15	11.0	26.6	26.8	26.70	3.6830	27.24
16	10.0	26.9	26.6	26.75	3.6760	27.05
17	9.0	26.7	27.2	26.95	3.6490	26.32
18	8.0	26.4	26.6	26.50	3.7110	28.00
19	7.0	26.6	25.8	26.20	3.7530	29.17
20	6.0	26.1	26.6	26.35	3.7320	28.58
21	5.0	26.6	26.5	26.55	3.7040	27.81
22	4.0	25.8	25.3	25.55	3.8490	31.91
23	3.0	26.6	26.4	26.50	3.7110	28.00
24	2.0	26.6	26.9	26.75	3.6760	27.05
25	1.0	26.3	26.6	26.45	3.7180	28.20

**Table 7:** Results of concrete ultrasound velocity test in borehole No. 6.

Ordinal Number	Height from the Bottom of the Floor (cm)	Ultrasound Netto Passing Time in Direction I-I *t_n I-I_* (μs)	Ultrasound Netto Passing Time in Direction II-II *t_n II-II_* (μs)	Mean Ultrasound Netto Passing Time *t_n_* (μs)	Ultrasound Wave Velocity *C_L_* (km/s)	Concrete Compression Strength *f_c_* (MPa)
1	21.0	47.5	49.5	48.54	2.122	26.90
2	20.0	49.4	51.0	50.20	2.051	20.50
3	19.0	60.4	62.0	61.21	1.682	5.14
4	18.0	46.7	47.0	46.84	2.199	35.20
5	17.0	46.7	47.6	47.12	2.185	33.70
6	16.0	47.4	48.5	47.96	2.147	29.52
7	15.0	46.7	47.7	47.16	2.184	33.49
8	14.0	47.6	47.1	47.35	2.175	32.50
9	13.0	48.1	47.4	47.77	2.156	30.42
10	12.0	46.7	46.7	46.71	2.205	35.92
11	11.0	46.8	46.3	46.55	2.212	36.82
12	10.0	47.9	46.5	47.20	2.182	33.28
13	9.0	48.9	47.6	48.25	2.134	28.18
14	8.0	49.6	47.7	48.66	2.116	26.38
15	7.0	48.4	47.7	48.07	2.142	29.00
16	6.0	46.2	47.6	46.91	2.195	34.82
17	5.0	48.8	45.2	46.99	2.192	34.39
18	4.0	50.4	46.2	48.34	2.130	27.78
19	3.0	47.4	47.2	47.27	2.179	32.92
20	2.0	45.7	46.6	46.16	2.231	39.09
21	1.0	45.7	45.7	45.66	2.255	42.18

**Table 8:** Results of concrete ultrasound velocity test in borehole No. 7.

Ordinal Number	Height from the Bottom of the Floor (cm)	Ultrasound Netto Passing Time in Direction I-I *t_n I-I_* (µs)	Ultrasound Netto Passing Time in Direction II-II *t_n II-II_* (µs)	Mean Ultrasound Netto Passing Time *t_n_* (µs)	Ultrasound Wave Velocity *C_L_* (km/s)	Concrete Compression Strength *f_c_* (MPa)
1	21.0	47.0	55.0	51.00	2.950	39.50
2	20.0	55.5	54.9	55.20	2.726	33.94
3	19.0	79.0	79.2	79.10	1.902	13.53
4	18.0	59.0	59.4	59.20	2.541	29.38
5	17.0	59.9	59.2	59.55	2.526	29.01
6	16.0	60.6	59.4	60.00	2.508	28.54
7	15.0	58.5	58.6	58.55	2.570	30.07
8	14.0	59.2	58.8	59.00	2.550	29.59
9	13.0	60.0	58.1	59.05	2.548	29.54
10	12.0	57.9	58.0	57.95	2.596	30.73
11	11.0	58.6	59.4	59.00	2.550	29.59
12	10.0	59.9	58.6	59.25	2.539	29.32
13	9.0	58.8	59.0	58.90	2.554	29.70
14	8.0	61.3	58.3	59.80	2.516	28.74
15	7.0	61.0	59.0	60.00	2.508	28.54
16	6.0	58.0	58.8	58.40	2.576	30.24
17	5.0	56.6	58.3	57.45	2.619	31.29
18	4.0	75.8	64.0	69.90	2.152	19.74
19	3.0	60.9	44.0	52.45	2.868	37.48
20	2.0	52.3	52.3	52.30	2.877	37.68
21	1.0	52.0	51.4	51.70	2.910	38.51

**Table 9:** Characteristics of strength gradients and measured strength values of the tested samples.

Ordinal Number	Characteristics of Floor Strength Distribution	Gradient of Strength Decrease in Near Surface Zone (MPa/cm)		Gradient of Strength Increase in Hardening Zone (MPa/cm)	Compressive Strength in the Entire Cross-Section (MPa)		
		∇*f_c,min_*	∇*f_c min,10 mm_*	∇*f_c,max_*	Max *f_c,max_*	Min *f_c,min_*	Average *f_c,av_*
1	Homogeneous with weakening of 10 mm of the top layer, (Table 2, Figure 8)	−16.7	−16.7	- *	33.38	12.48	28.42
2	Homogeneous with weakening of 35 mm of the top layer (Table 3, Figure 9)	−3.8	−6.9	- *	45.49	24.24	35.29
3	Variable across the entire cross-section with quick weakening of 50 mm of the top layer (Table 4, Figure 10 and Figure 15)	−2.8	−7.6	- *	30.97	8.78	22.55
4	Surface strengthened to the designed value of 30 MPa from 22 MPa (Table 5, Figure 11)	−9.0	−9.0	7.1	41.14	23.22	33.45
5	Poorly hardened surface with low strength (Table 6, Figure 12)	−4.3	−5.1	2.4	31.91	16.33	23.09
6	Hardened surface with too low strength (Table 7, Figure 13)	−30.1	−30.1	10.9	42.18	5.14	30.86
7	Excessively hardened surface with low strength (Table 8, Figure 14)	−15.8	−15.8	13.0	39.5	13.53	30.22

*—floor without surface hardening.
